# Prevalence of Sleep Apnea in Patients with Syncope of Unclear Cause: SINCOSAS Study

**DOI:** 10.3390/medicina61050887

**Published:** 2025-05-13

**Authors:** María-José Muñoz-Martínez, Alberto Fernández-Villar, Manuel Casal-Guisande, Enrique García-Campo, Dolores Corbacho-Abelaira, Ana Souto-Alonso, Bernardo Sopeña

**Affiliations:** 1 Pulmonary Department, Hospital Universitario Álvaro Cunqueiro, 36312 Vigo, Spain; 2 NeumoVigo I+i Research Group, Galicia Sur Health Research Institute (IIS Galicia Sur), SERGAS-UVIGO, 36312 Vigo, Spain; 3 Faculty of Medicine and Dentistry, University of Santiago de Compostela, 15782 Santiago de Compostela, Spain; 4 Centro de Investigación Biomédica en Red, CIBERES ISCIII, 28029 Madrid, Spain; 5 School of Industrial Engineering, University of Vigo, 36310 Vigo, Spain; 6 Fundación Pública Galega de Investigación Biomédica Galicia Sur, Hospital Álvaro Cunqueiro, 36312 Vigo, Spain; 7 Cardiology Department, Hospital Universitario Álvaro Cunqueiro, 36312 Vigo, Spain; 8 Pulmonary Department, Hospital Ribera Povisa, 36211 Vigo, Spain; 9 Pulmonary Department, Hospital Universitario de A Coruña, 15006 A Coruña, Spain; 10 Internal Medicine Department, Hospital Clínico Universitario de Santiago de Compostela, 15782 Santiago de Compostela, Spain

**Keywords:** sleep apnea, syncope, heart rate variability

## Abstract

*Background and Objectives:* The association between syncope and sleep apnea (SA) has been scarcely investigated. Dysfunction of the autonomic nervous system (ANS) may represent a shared pathophysiological mechanism. This study aimed to determine the prevalence of SA in patients with syncope of unclear cause (SUC), identify potential associated factors, and evaluate nocturnal heart rate variability (HRV) as a marker of ANS function. *Materials and Methods:* A prospective cohort study was conducted in adult patients diagnosed with SUC. Nocturnal cardiorespiratory polygraphy was performed to detect the presence of SA. A range of variables potentially associated with SA was collected. Both SA diagnosis and HRV parameters were assessed using the Embletta^®^ MPR polygraph system. *Results:* A total of 156 patients were enrolled (57% male), with a mean age of 64 years and a mean body mass index of 27.5 kg/m^2^ (range: 24.8–32.2). Hypertension was present in 46% of the cohort. The overall prevalence of SA was 78.2% (95% CI: 71.7–84.4%), with 28.7% classified as severe. Age (OR = 1.04; 95% CI: 1.01–1.07) and BMI (OR = 1.17; 95% CI: 1.06–1.28) were independent predictors of SA. Mean RR interval was significantly lower in patients with SA compared to those without (942 ms vs. 995 ms; *p* = 0.04). No significant differences in HRV parameters were observed between the two groups. *Conclusions:* This study found a high prevalence (nearly 78%) of SA among adult patients with SUC, particularly in individuals over 50 years of age and those who were overweight. However, this association could not be predicted based on clinical variables alone. No significant differences in nocturnal HRV were detected between patients with SUC with and without SA.

## 1. Introduction

Syncope is defined as a transient loss of consciousness resulting from cerebral hypoperfusion. It is characterized by a sudden onset, brief duration, and complete spontaneous recovery. The primary pathophysiological mechanisms underlying syncope often involve autonomic nervous system (ANS) dysfunction, which can trigger hypotension, bradycardia, and subsequent loss of consciousness [1,2].

Similarly, sleep apnea (SA) is defined by recurrent episodes of complete or partial upper airway obstruction during sleep, leading to micro-arousals, oxygen desaturation, and fluctuations in intrathoracic pressure. These events can activate intermediate pathophysiological pathways, including ANS dysfunction [3].

Given this shared involvement of the ANS, heart rate variability (HRV) is considered one of the most reliable non-invasive methods to assess ANS activity [4,5]. HRV reflects the oscillations in the intervals between consecutive heartbeats (RR intervals on the electrocardiogram), indicating the body’s ability to adapt to and recover from various stimuli. High HRV is typically associated with a healthy, responsive autonomic system, whereas low HRV is indicative of ANS dysfunction. In fact, reduced HRV has been shown to predict all-cause mortality in a meta-analysis involving nearly 40,000 individuals, both healthy and ill [6].

Over recent decades, a pathophysiological link between ANS dysfunction and several clinical entities has been established, including cardiovascular disease [7], sleep disorders [8], and pulmonary hypertension [9]. This growing body of evidence reinforces the relevance of ANS assessment in a wide range of conditions.

In a small cohort of 12 patients with both syncope and SA, Puel et al. [10] suggested that sleep fragmentation, significant intrathoracic pressure swings, and intermittent hypoxia may contribute to ANS dysfunction, thereby promoting vagal manifestations during wakefulness. In this group, treatment with continuous positive airway pressure (CPAP) reduced the frequency of syncopal episodes after 6 months. Building on these findings, subsequent observational studies have also supported associations between reduced HRV and the presence of syncope [11] as well as SA [8,12].

Both syncope and SA are highly prevalent conditions that significantly affect quality of life and place a considerable burden on healthcare systems [13,14,15,16]. While many studies have linked SA to arrhythmias, ischemic heart disease, and sudden cardiac death [17,18,19,20,21], the potential interplay between SA and syncope has received little attention. Additionally, it is unclear whether HRV can serve as a useful tool for identifying patients with syncope who are at increased risk of having SA.

In light of these considerations, this study was therefore designed to (1) determine the prevalence of SA in patients with syncope of unclear cause (SUC), and (2) evaluate whether clinical characteristics and HRV parameters can help identify which patients with syncope might benefit from undergoing cardiorespiratory polygraphy to assess the presence of SA.

## 2. Materials and Methods

### 2.1. Study Population and Procedures

#### 2.1.1. Study Design and Eligibility Criteria

This was a multicenter, prospective cohort study conducted at three tertiary care centers in northwestern Spain: Álvaro Cunqueiro Hospital and Ribera Povisa Hospital in Vigo, and the Complexo Hospitalario Universitario de A Coruña. The recruitment period extended from June 2019 to May 2024.

We included adult patients (>18 years) diagnosed with SUC following a comprehensive cardiological and neurological assessment, in accordance with current clinical guidelines [1]. Patients were recruited from Cardiology departments—primarily those on the waiting list for implantable Holter monitoring—as well as Neurology and Emergency Departments.

Exclusion criteria were previously diagnosed with SA, epilepsy, or illicit drug use.

#### 2.1.2. Clinical Data Collection

For each patient, demographic and anthropometric variables were recorded, including age, sex, and body mass index (BMI). Clinical history encompassed cardiovascular diseases (such as ischemic or valvular heart disease, hypertension, arrhythmias, and stroke), respiratory conditions (asthma and chronic obstructive pulmonary disease), diabetes mellitus, and dyslipidemia. Lifestyle information included smoking status and cumulative exposure, expressed in pack-years. Sleep-related symptoms—such as excessive daytime sleepiness (assessed using the Epworth Sleepiness Scale), nocturnal awakenings, and daytime fatigue—were also collected. In addition, the number of syncopal episodes reported during the previous year was documented, along with the use of negative inotropic or cardiodepressant drugs potentially capable of reducing heart rate. BMI was calculated by dividing weight in kilograms by height in meters squared and classified into the following categories: normal (<25 kg/m^2^), overweight (25–29.9 kg/m^2^), obesity grade I (30–34.9 kg/m^2^), grade II (35–39.9 kg/m^2^), and grade III or morbid obesity (≥40 kg/m^2^).

#### 2.1.3. Respiratory Polygraphy and Sleep Apnea Diagnosis

All participants underwent home-based nocturnal respiratory polygraphy using the Embletta^®^ MPR system, which enables synchronized electrocardiographic monitoring and automated analysis of HRV. The Embletta^®^ MPR polygraph used in this study was equipped with electrocardiographic monitoring, a thermistor channel, nasal airflow sensor, thoracic and abdominal respiratory effort bands, and a fingertip pulse oximeter. HRV was assessed using an automated report. Respiratory events were first detected through automated analysis and subsequently reviewed and manually corrected throughout the entire recording.

Recordings were performed from 12:00 a.m. to 7:00 a.m. The polygraphy provided the following parameters: the apnea-hypopnea index (AHI), total time with oxygen saturation below 90% (TC90), desaturation index ≥ 3% (ID3), and the number of obstructive, central, and mixed apneas, as well as the number of hypopneas. SA was diagnosed according to the 2021 clinical guidelines of the Spanish Society of Pulmonology and Thoracic Surgery (SEPAR) [3]. Diagnosis was confirmed when one of the following conditions was met: (1) an AHI ≥ 15 events per hour, predominantly obstructive in nature; or (2) an AHI ≥ 5 events per hour accompanied by at least one of the following: excessive daytime sleepiness, non-restorative sleep, fatigue, or deterioration in sleep-related quality of life not attributable to other causes.

The AHI was calculated as the total number of apneas plus the total number of hypopneas, divided by the total recording time (please, see Equation (1)).

(1) $$AHI=\frac{number\;of\;apneas+number\;of\;hypopneas}{recording\;time}$$

The respiratory events included in the AHI are apneas and hypopneas, defined as follows:

- Obstructive apnea: ≥90% reduction in airflow lasting at least 10 s, with continued respiratory effort.
- Central apnea: ≥90% reduction in airflow for at least 10 s, without respiratory effort.
- Mixed apnea: initially presents without respiratory effort (as a central apnea), followed by resumption of effort.
- Hypopnea: ≥30% reduction in airflow for at least 10 s, associated with a ≥3% oxygen desaturation.

According to SEPAR guidelines [3], central SA was diagnosed when the AHI was ≥5 events/h and more than 50% of apneas were central.

AHI was used to classify SA severity as follows:

- Normal: AHI < 5 events/h.
- Mild: 5 ≤ AHI < 15 events/h.
- Moderate: 15 ≤ AHI < 30 events/h.
- Severe: AHI ≥ 30 events/h.

#### 2.1.4. Heart Rate Variability Assessment

HRV was analyzed using the electrocardiographic signal recorded by the Embletta^®^ MPR system, in 5-min epochs over a 7-h nighttime monitoring period. The following parameters were assessed: for time-domain analysis, RR average (mean RR interval), SDNN (standard deviation of all RR intervals), SDANN (standard deviation of average RR intervals in 1-min segments), RMSSD (root mean square of successive differences), SDNN index (mean of the standard deviations of all RR intervals for each 1-min segment), NN50 (number of successive RR intervals differing by more than 50 ms), pNN50 (percentage of NN50), and the triangular index (total number of RR intervals divided by the height of the histogram peak). For frequency-domain analysis, total power (overall variance of RR intervals), high-frequency (HF) power, low-frequency (LF) power, and very low-frequency (VLF) power were calculated. According to European standards, HRV was considered reduced when time-domain indices such as SDNN, SDANN, RMSSD, NN50, pNN50, the SDNN index, triangular index, or HF power (indicative of parasympathetic activity) were decreased, and/or when VLF or LF power (reflecting sympathetic activity) were increased. The RR average was interpreted as an indicator of baseline heart rate [4].

#### 2.1.5. Ethical Considerations

The study was approved by the Clinical Research Ethics Committee of Galicia (protocol number: 2019/048). All participants received detailed information about the study and signed written informed consent before inclusion.

### 2.2. Statistical Analysis

Continuous variables were summarized as medians and interquartile ranges (25th–75th percentiles), while categorical variables were presented as absolute and relative frequencies (%). The prevalence of SA was reported with its corresponding 95% confidence interval (95% CI).

Between-group comparisons were made using the Mann–Whitney U test for continuous variables and Chi-square or Fisher’s exact test for categorical variables. A *p*-value < 0.05 was considered statistically significant.

Variables that were significantly associated with the presence of SA in univariate analysis were included in a multivariate logistic regression model using forward stepwise selection to identify independent predictors. Results were expressed as odds ratios (OR) with 95% confidence intervals.

All statistical analyses were performed using SPSS for Windows, version 25.0 (IBM Corp., Armonk, NY, USA). The sample size was recalculated after an interim analysis of the first 50 patients, in whom SA prevalence was estimated at approximately 70%. For a 95% confidence level and a 5% margin of error, the final sample size was adjusted accordingly. Additionally, based on the findings of a meta-analysis by Guo et al. [8], it was estimated that a sample of at least 120 patients with SA would be required to detect differences in HRV parameters with 80% power and a 5% significance level. Therefore, the final sample of 156 patients, including 122 with SA, was considered sufficient to meet the primary objective.

## 3. Results

### 3.1. Patient Characteristics

A total of 156 patients with SUC were included in the study, of whom 89 were male (57%). The mean age at inclusion was 64 years (range: 52–74 years). Among the total cohort, 122 patients [78% (95% CI: 71.7–84.4%)] were diagnosed with SA. Demographic, anthropometric, and clinical characteristics for all participants, as well as stratified comparisons between those with and without SA, are presented in Table 1.

### 3.2. Comparison Between Patients with and Without Sleep Apnea

Patients with SUC and SA were significantly older than those without SA. In individuals under 50 years of age, 17 out of 32 (53%) had SA. This prevalence increased markedly in older age groups, reaching 82% (56/68) in patients aged 50–69 years and 87% (49/56) in those older than 70 years.

BMI was also significantly higher in the SA group, and a linear association was observed between increasing BMI and SA prevalence. Among 41 patients with a BMI < 25, 24 (58%) had SA; this proportion rose to 83% among overweight individuals and 100% among those with any degree of obesity.

Cardiovascular risk factors—including hypertension, diabetes, dyslipidemia—and smoking exposure (pack-years) were significantly more prevalent in the SA group. Although ischemic heart disease and atrial fibrillation (AF) appeared more frequently in patients with SA (AF: 20% vs. 6%), these differences did not reach statistical significance, possibly due to the limited sample size (AF *p* = 0.05).

Interestingly, none of the typical symptoms of SA—such as daytime sleepiness, fatigue, or nocturnal awakenings—nor the Epworth Sleepiness Scale score differed significantly between patients with and without SA.

In the multivariate logistic regression analysis, only age and BMI remained independently associated with the presence of SA. Older age (OR = 1.04; 95% CI: 1.01–1.07) and higher BMI (OR = 1.17; 95% CI: 1.06–1.28) were significantly predictive of SA in patients with syncope.

### 3.3. Respiratory Polygraphy Findings

Among the 122 patients diagnosed with SA, 59 (48.4%) had mild SA, 28 (23%) moderate, and 35 (28.7%) severe. No patients were identified with predominantly central SA, nor was Cheyne–Stokes respiration detected. Regarding therapeutic approach, 70 patients (61.4%) were prescribed CPAP, while one patient (0.8%) received a positional therapy device, and four (3.2%) were treated with a servo-ventilator. The remaining patients were advised to implement positional and lifestyle-related (hygienic-dietary) measures only. Table 2 and Table 3 provide a detailed analysis of respiratory parameters in the overall study population and in a subgroup of patients not receiving negative inotropic drugs, stratified by the presence or absence of SA.

### 3.4. Heart Rate Variability Analysis

HRV analysis was completed in all patients. The mean RR interval was significantly lower in the SA group compared to those without SA. The average HF power displayed a discordant pattern, and the rest of the HRV parameters showed no relevant intergroup differences (Table 2).

Notably, the use of negative inotropic medications—including amitriptyline, doxazosin, flecainide, propranolol, atenolol, and amiodarone—did not significantly affect HRV measurements. No differences were found in HRV indices when comparing the 102 patients not receiving these agents to those who were (Table 3).

No nocturnal arrhythmias were detected upon manual analysis of the electrocardiogram.

## 4. Discussion

To our knowledge, this is the first study to assess the prevalence of SA in patients with SUC, analyzing associated clinical factors and HRV findings. The present results show that SA was undiagnosed in 78% of patients with SUC, and that approximately 30% had severe SA. The potential relationship between syncope and SA has received little attention in the literature. Only the retrospective study by Valencia et al. [22] reported a higher frequency of neurally mediated syncope in patients with severe SA compared to controls without SA (30% vs. 14%, OR = 3.1; *p* < 0.05).

Although SA is a frequent condition, its estimated prevalence in the general population varies widely depending on the diagnostic criteria, methodology, and population characteristics. In 2020, Benjafield et al. [13] published a global estimate of SA prevalence based on population-based studies from various continents, including Spain, reporting a range between 4% and 30%. In contrast, the 78% prevalence observed in our cohort is markedly higher. This difference may be explained by the demographic and clinical characteristics of our population, which included older adults with higher BMI and greater prevalence of hypertension and atrial fibrillation—risk factors also associated with SA. In fact, the high prevalence of SA in our cohort aligns more closely with that observed in high-risk subpopulations, such as patients with resistant hypertension (up to 80%) [23] and atrial fibrillation (around 50%) [18], which prompted a change in the clinical management of these patients.

In our analysis, age and BMI were the only independent predictors of SA in patients with SUC. This finding is consistent with known risk factors for SA in the general population [24]. However, the observed odds ratio was relatively low, a finding that may be explained by the small differences in mean age and BMI between the two groups. Syncope exhibits a bimodal age distribution, with one peak occurring between 15 and 20 years of age (a group underrepresented in our sample) and another around 60 years [25]. According to the literature [26], the prevalence of SA increases linearly with age, reaching a plateau around 65 years. This, combined with the fact that cardiologic causes are the most common etiology of syncope in older adults [27], may explain why the average age of patients with SUC in our cohort was close to 60 years. Regarding BMI, obese individuals tend to have higher cardiovascular risk, making the cause of their syncope more readily identifiable [28]. Therefore, the predominance of overweight rather than obese individuals in our cohort aligns with the diagnosis of SUC but makes the high prevalence of SA less expected. Notably, in patients over 50 years of age and in those with a BMI >25, the prevalence of SA exceeded 80%. Even among younger, non-obese individuals, the prevalence of SA remained substantially higher than expected based on population studies, further supporting the relevance of systematic SA screening in patients with unexplained syncope. This pattern is consistent with findings from other clinical populations where relevant conclusions were drawn from samples of similar or smaller sizes. For example, Pedrosa et al. [29] demonstrated a 64% prevalence of SA in 125 patients with resistant hypertension, and Logan et al. [23] reported an 83% prevalence in just 41 patients. These studies support the adequacy of our sample size for initial characterization of the SUC–SA association.

Interestingly, patients with SUC and SA did not present with greater daytime sleepiness or other classical SA symptoms compared to those without SA. This asymptomatic presentation could be related to heightened sympathetic tone (this hypothesis could not be demonstrated in our study), a phenomenon described in patients with SA associated with cardiovascular disease [30,31,32], which may mask typical symptoms. Consequently, relying solely on symptom-based screening tools like the Epworth Sleepiness Scale may lead to underdiagnosis in this population. It is worth noting that, despite the higher prevalence of cardiovascular risk factors, most patients in our cohort were clinically asymptomatic with respect to suspected SA. Neither typical symptoms (such as snoring, witnessed apneas, or daytime sleepiness), nor elevated scores on the Epworth Sleepiness Scale, nor the coexistence of cardiovascular risk factors, facilitated a prior diagnosis of SA.

Contrary to most epidemiological studies, SA in our cohort was not more prevalent in men. This discrepancy may be attributed to the higher mean age of female participants, as SA prevalence increases in postmenopausal women, reaching rates similar to those in men [24].

In our cohort, no cases of predominantly central SA or Cheyne–Stokes respiration were observed. This is likely due to the inclusion of patients with syncope of unclear cause, whereas central SA is typically associated with identifiable cardiologic etiologies [33].

BMI and age have been described in the literature [34,35] as factors that influence autonomic nervous system function, which could represent a potential mechanism linking them to syncope. However, current evidence is insufficient to establish a causal relationship.

Regarding autonomic function, our analysis of HRV parameters did not reveal consistent differences between patients with and without SA. Only the mean RR interval was significantly lower in the SA group, reflecting a lower heart rate rather than altered HRV. Regarding the RR interval, it is not considered a true HRV parameter; its primary significance lies in the fact that a lower RR reflects an increased nocturnal heart rate [36]. In this context, the reduction in RR could be secondary to SA events; however, further studies are needed to confirm whether this decrease can indeed be attributed to respiratory disturbances such as apneas. This finding aligns with that of Zhu et al. [37], who also described reduced nocturnal heart rate in SA patients. Although several studies and meta-analyses [12,38] have demonstrated reduced parasympathetic tone and increased sympathetic responsiveness in SA—particularly in frequency-domain HRV measures—our study did not reproduce those associations. These discrepancies may be due to methodological differences, including sample size, home-based recording conditions, and nocturnal versus daytime HRV analysis.

Previous studies have explored HRV alterations in patients with vasovagal or unexplained syncope [10,39,40]. While some suggest increased sympathetic activity or reduced HRV, these analyses did not evaluate the potential contribution of SA to autonomic imbalance. Cintra et al. [40], for example, reported contradictory HRV findings in vasovagal syncope, with reduced time-domain HRV measures but also decreased low-frequency power during REM sleep—suggesting altered sympathetic-parasympathetic balance without consistent changes in daytime HRV.

Although HRV parameters did not differ significantly between groups, the AHI and the number of respiratory events—both key components in the definition of SA—were, as expected, elevated in patients with SA. These findings, together with the increased TC90, elevated ID3, and reduced RR interval, might suggest that repeated and sustained oxygen desaturation events, along with a tendency toward nocturnal tachycardia, play a role in the pathophysiology of syncope in this patient cohort. As suggested in our study, greater AHI severity appears to be associated with increased nocturnal heart rate (lower RR). However, these observations do not allow us to conclude that nocturnal respiratory events are the direct cause of daytime syncopal episodes.

Our study has several limitations. First, we used home-based respiratory polygraphy rather than full polysomnography, which is considered the gold standard for SA diagnosis. Second, the analysis of HRV was limited by the absence of validated reference values for nocturnal recordings and the potential influence of uncontrolled external factors (e.g., stress, insomnia, environmental temperature, or caffeine intake). Additionally, the sample size may have been insufficient to detect subtle but clinically relevant differences in HRV parameters between groups. However, the final sample size is consistent with prior power calculations based on literature estimates. Specifically, our calculation was supported by data from a meta-analysis by Guo et al. [8], which indicated that approximately 120 patients with SA would be needed to detect significant HRV differences with 80% power and a 5% significance level. This supports the adequacy of our cohort for the study’s primary aim.

The high proportion of patients with SA resulted in an imbalance between groups, with a predominance of SA patients compared to those without SA, which limits the value of between-group comparisons. Although no significant differences were found in the univariate and multivariate analyses, the higher prevalence of cardiovascular risk factors in the SA group may have acted as a confounding variable.

Nevertheless, our findings have potential implications for clinical practice. Several case reports and small series [10,41] have suggested that CPAP therapy in patients with syncope and SA may reduce the frequency of syncopal episodes. Early identification and treatment of SA in patients with SUC could therefore have diagnostic and therapeutic value and might even reduce the need for invasive procedures such as implantable long-term cardiac monitors.

The SINCOSAS study remains ongoing, with patient recruitment still in progress. Future phases aim to include a control group (patients without syncope) and to conduct long-term follow-up to assess the impact of SA treatment on the frequency of syncopal episodes and changes in HRV.

## 5. Conclusions

In conclusion, our study shows that SA is highly prevalent in patients with syncope of unclear cause, particularly in those over 50 years of age and with elevated BMI. While no clear HRV patterns were identified in this population, the data support the need for increased clinical suspicion and routine respiratory polygraphy in the diagnostic work-up of patients with unexplained syncope. This approach may improve diagnostic yield, guide therapeutic decisions, and potentially reduce the need for invasive procedures such as implantable cardiac monitors.

## Figures and Tables

**Table 1:** Variables collected in all patients and differentiation according to diagnosis or not of SA.

Variables	Total N = 156	No SA N = 34	With SA N = 122	Value of *p*
Sex male	89 (57.1%)	16 (47.7%)	73 (59.8%)	0.80
Age (years)	64 (52.2–74)	52.5 (42–69)	65 (55.7–75)	0.00
BMI (kg/m^2^)	27.5 (24.8–31.2)	25.1 (22.1−28.9)	28 (25.7–32.1)	0.00
Tobacco * Never smoker Ex-smoker Active smoker	70 (45.2%) 53 (34.2%) 32 (20.6%)	13 (38.2%) 11 (32.4%) 10 (29.4%)	57 (47.1%) 42 (34.7%) 22(18.2%)	0.34
Packs/years (in smokers and ex-smokers)	25.5 (10–51)	12 (4.5–34.5)	30 (17.57)	0.01
Ischaemic heart disease	18 (8.6%)	2 (5.9%)	16 (13.2%)	0.36
Valvular heart disease	3 (1.9%)	1 (2.9%)	2 (1.7%)	0.52
Atrial fibrillation	26 (16.9%)	2 (5.9%)	24 (20%)	0.05
High Blood Pressure (HBP)	71 (46.1%)	8 (23.5%)	63 (53.5%)	0.00
Stroke	1 (0.6%)	0 (0%)	1 (0.8%)	0.99
Diabetes	22 (14.1%)	1 (2.9%)	21 (17.2%)	0.04
Dyslipidaemia	59 (37.8%)	6 (17.6%)	53 (43.4%)	0.01
Chronic obstructive pulmonary disease (COPD)	7 (4.5%)	0 (0%)	7 (5.7%)	0.34
Asthma	13 (8.3%)	5 (14.7%)	8 (6.6%)	0.16
Epworth Scale	7 (3–13)	8 (3–14)	7 (3–13)	0.71
Daytime fatigue	80 (51.3%)	14 (44.1%)	65 (53.3%)	0.34
Nocturnal awakenings	78 (50%)	16 (47.1%)	62 (50.8%)	0.69
Lack of concentration	58 (37.2%)	11 (32.4%)	47 (38.5%)	0.51
Apneas observed	38 (24.4%)	9 (26.5%)	29 (23.8%)	0.74
No of syncope/previous year	3 (1.4)	2 (1–5)	3 (1–4)	0.95

* In the tobacco category, for the total and with SA, calculations have been performed for 155 and 121 individuals respectively, as there is one missing patient.

**Table 2:** Respiratory parameters and parameters related to HRV of the total sample and differentiated according to the presence of SA or not.

Variables	Total N = 156	No SA N = 34	With SA N = 122	Value of *p*
AHI	9.4 (5.5–28.1)	1.65 (0.97–3.1)	15.8 (8.27–31.97)	0.00
ID3	10.2 (4.8–25.5)	1.5 (0.6–2.29)	16.3 (8.2–30.92)	0.00
TC90	2.1 (0.1–9.5)	0 (0–0.52)	4.6 (0.6–13.02)	0.00
N obstructive apneas	4 (1–25)	0.1 (0–3.25)	6.5 (1.7–46.25)	0.00
N central apneas	0.5 (0–4)	0 (0–1)	1 (0–4.25)	0.00
N mixed apneas	0 (0–1)	0 (0–2)	0 (0–0)	0.01
N hypoapneas	53 (24.2–104.7)	6 (3–13)	65 (42.5–123.25)	0.00
Average RR (ms)	953 (844–1034)	995 (876–1187)	942 (829–1022)	0.04
SDNN (ms)	102.5 (78–134.7)	101 (78.7–128)	102.5 (77.7–156)	0.69
SDNN index (ms)	74 (51–103)	75.5 (48–92.5)	73.5 (51–113)	0.40
RMSSD (ms)	70.5 (42–123.5)	68.5 (44.25–102.5)	70.5 (41.7–148)	0.43
NN50	2279 (640–6775.7)	2307 (629.5–7071.5)	2278.5 (629–6707.2)	0.70
%NN50	11.5 (3.2–31.1)	11.9 (4.1–27.9)	10.2 (3.2–32.1)	0.88
SDANN (ms)	61 (43–111)	72 (46.5–117)	59.5 (42–108.7)	0.41
Average total power (ms^2^)	21,144 (13,495–37,146)	25,996.5 (12,613.5–45,254.5)	20,769 (13,598–34,743.5)	0.64
Average VLF power (ms^2^)	9465.5 (3586.2–19,477.5)	9612 (4536.5–21,327.5)	9426 (3341.5–19,136.5	0.57
Average LF power (ms^2^)	7512.5 (3822–12,411.5)	8144 (4453–16,169.5)	7271 (3721–11,417)	0.53
Average HF power (ms^2^)	3597 (2334–5410)	4779 (1457.7–6388)	3306 (2459.5–5125.5)	0.25
LF/HF	1.9 (1.1–3.3)	2 (1.2–3.5)	1.9 (1.1–3.2)	0.89
Triangular index HRV	16 (11–20)	14 (9.7–20.2)	16 (11–20)	0.45

**Table 3:** Respiratory parameters and parameters related to HRV differentiated according to the presence or absence of SA, excluding patients with negative inotropic drugs.

Variables	No SA N = 25	With SA N = 77	Value of *p*
AHI	1.5 (0.7–2.1)	12.6 (7.7–28.35)	0.00
ID3	1.4 (0.6–2.55)	12.6 (81–25.75)	0.00
TC90	0 (0–2.5)	4.8 (0.7–15.1)	0.00
Number obstructive apneas	1 (0–3.5)	6 (1–52)	0.00
Number Central apneas	0 (0–1)	1 (0–3.5)	0.01
Number mixed apneas	0 (0–0)	0 (0–1)	0.01
Number hypoapneas	5 (2.5–9)	63 (40.5–107)	0.00
Average RR (ms)	992 (875–1179)	937 (827.7–1005)	0.02
SDNN (ms)	104 (80–129)	99.5 (73–125.2)	0.50
SDNN index (ms)	79 (51–93)	69.5 (48.5–99.5)	0.54
RMSSD (ms)	73 (48–97.5)	62.5 (41–104.5)	0.54
NN50	3067 (838–7437)	1930.5 (591.7–4760.7)	0.59
%NN50	12.2 (4.6–32)	8 (2.5–22.1)	0.93
SDANN (ms)	79 (53–117)	59 (42–108)	0.84
Average total power (ms^2^)	25,366 (11,885–50,560)	27,237 (16,073.5–37,825.5)	0.97
Average VLF power (ms^2^)	9544.5 (4603.7–21,817)	11,958 (7227–20,937.5)	0.53
Average LF power (ms^2^)	8237 (4321.7–17,930)	9221 (5019.5–13,033.5)	0.81
Average HF power (ms^2^)	4870 (1568.5–6113)	3295 (2271.5–5099.5)	0.22
LF/HF	2.4 (1.3–3.6)	2.2 (1.45–3.6)	0.69
Triangular index HRV	16 (9.5–20.5)	16 (12–18.5)	0.79

## Data Availability

The data presented in this study are available on request from the corresponding author.
